# Suppressing of Power Line Artifact From Electroencephalogram Measurements Using Sparsity in Frequency Domain

**DOI:** 10.3389/fnins.2021.780373

**Published:** 2021-10-28

**Authors:** Jin-Lin Tan, Zhi-Feng Liang, Rui Zhang, You-Qiang Dong, Guang-Hui Li, Min Zhang, Hai Wang, Na Xu

**Affiliations:** ^1^School of Aerospace Science and Technology, Xidian University, Xi’an, China; ^2^Shaanxi Aerospace Technology Application Research Institute Co., Ltd, Xi’an, China; ^3^The Second Affiliated Hospital of Xiamen Medical College, Xiamen, China

**Keywords:** EEG, brain imaging, sparse representation, noise reduction, salient feature enhancement

## Abstract

Electroencephalogram (EEG) plays an important role in brain disease diagnosis and research of brain-computer interface (BCI). However, the measurements of EEG are often exposed to strong interference of power line artifact (PLA). Digital notch filters (DNFs) can be applied to remove the PLA effectively, but it also results in severe signal distortions in the time domain. To address this problem, spectrum correction (SC) based methods can be utilized. These methods estimate harmonic parameters of the PLA such that compensation signals are produced to remove the noise. In order to ensure high accuracy during harmonic parameter estimations, a novel approach is proposed in this paper. This novel approach is based on the combination of sparse representation (SR) and SC. It can deeply mine the information of PLA in the frequency domain. Firstly, a ratio-based spectrum correction (RBSC) using rectangular window is employed to make rough estimation of the harmonic parameters of PLA. Secondly, the two spectral line closest to the estimated frequency are calculated. Thirdly, the two spectral lines with high amplitudes can be utilized as input of RBSC to make finer estimations of the harmonic parameters. Finally, a compensation signal, based on the extracted harmonic parameters, is generated to suppress PLA. Numerical simulations and actual EEG signals with PLA were used to evaluate the effectiveness of the improved approach. It is verified that this approach can effectively suppress the PLA without distorting the time-domain waveform of the EEG signal.

## Introduction

In the measurement of Electroencephalogram (EEG), differences in voltages from distinct sites of the brain are recorded over a period of time (Hernández-Ronquillo et al., 2020; Sun et al., 2020). The EEG signal contains abundant information related to physiological, psychological and pathological activities of the brain. Therefore, the analysis of EEG signals is of vital importance in the clinical diagnosis and treatment of many diseases, such as Alzheimer’s disease (Faiman et al., 2021), depression (Miao et al., 2021), idiopathic epilepsy and psychogenic non-epileptic seizures (Sharma G. et al., 2021). On the other hand, EEG also plays an important role in the research of brain-computer interface (BCI). BCI is referred to as a non-invasive way of human-machine interface between the brain and exterior devices (Dagdevir and Tokmakci, 2021). BCI does not rely on conventional neurotransmission pathways (Deshpande et al., 2017). Nowadays, many brain imaging methodologies have been adopted by the researchers, such as EEG, magnetic resonance imaging (MRI) and functional MRI (fMRI; Hong et al., 2018). Among the above mediums, EEG is regarded as the most convenient one due to its mobility and lower cost.

In medical applications, owing to non-stationary characteristics of EEG features and differences among individual measurements, the analysis of EEG signals is comparatively complicated and difficult (An et al., 2021). The traditional signal analysis method based on fast Fourier transform (FFT) cannot meet the requirements of EEG signal analysis. In order to reduce excessive professional requirements for medical staff to identify EEG, deep learning (DL) based methods, such as convolutional neural network (CNN; Cao et al., 2019; Prathaban and Balasubramanian, 2021), are introduced to ensure intelligent understanding of EEG signals, especially for large scale datasets. Aiming at achieving robust recognition ability, researchers have designed novel DL neural networks with distinguished types of network architecture, input formulation, and activation function (Saegh et al., 2021). Li proposed an FFT-based deep feature learning method for EEG classification (Li and Chen, 2021). Shankar proposed a DL based epileptic seizure detection algorithm by using 2D recurrence plot images generated from EEG signals for specific brain rhythms (Shankar et al., 2021). Jana studied an efficient seizure prediction technique based on the combination of CNN and a novel technique of channel reduction (Jana and Mukherjee, 2021). According to the above studies, researchers also attached significant attentions in deriving special features from raw EEG signals because it can help improve the accuracy and efficiency of the DL neural networks.

In spite of the versatility of EEG signal in clinical applications and brain imaging science, it is reported that the salient features within the EEG signals are weak in energy. On the other hand, a variety of artifacts are likely to be incorporated during acquisitions of EEG signals. To investigate the essential physiological information corrupted by these noisy artifacts, pre-processing based on signal processing techniques is indispensable. Among the many sources of interference, the power line artifact (PLA), at the frequency of 50 or 60 Hz, is almost inevitable in “field” or mobile EEG measurements outside the lab (Leske and Dalal, 2019). The presence of PLA can significantly affect the analysis and extraction of essential features from EEG recordings. Although shielding of the environment has been recognized as an effective way to reduce PLA in the lab, it is often impractical in scenarios of natural environments. Therefore, alternative solutions, hardware based or software based, must be adopted to address this PLA problem. In comparison, the advantages of software-based solutions are more prominent because they are more flexible and easier to be realized. In state-of-the-art researches, signal processing tools, such as digital notch filters (DNFs; Piskorowski, 2013), short time Fourier transform (STFT; Huang et al., 2019), wavelet transform (WT; He et al., 2015; Huang et al., 2021; Sharma S. et al., 2021), and empirical mode decomposition (EMD; Taran et al., 2018), have been utilized to remove the PLA. However, according to theoretical investigations, the above techniques can be interpreted as digital filters with specific passing band in the frequency domain (Wu and Huang, 2004). Owing to the problems of energy leakage and picket-fence effect (PFE) in canonical Fourier transform, such kinds of digital filters will cause non-ignorable distortions in filtered results, whereas the same problems also occur in hardware-based solutions. To overcome this side effect, a feasible way is to construct a compensation signal with high precision. According to engineering experiences, PLA can be modeled as a sinusoidal component consisting of a simple harmonic wave. While, a sinusoidal component can be uniquely determined by harmonic parameters of amplitude, frequency and phase. Therefore, to retrieve the harmonic parameters become the essential task in PLA removal.

Sparse representation (SR) is a comparatively new development of signal expansion (Li and Chen, 2008). It expresses an input signal in terms of linear combinations of atoms from a dictionary. It has achieved tremendous successes in various engineering applications (Wang et al., 2016; Yang et al., 2020; Zhang et al., 2021). Conventional SRs are implemented using iterative algorithms and may require large computational resources (Cao et al., 2021; Chen et al., 2021; An et al., 2022). If the efficiency of SR algorithm can be improved, it will effectively expand the scope of its use in medical engineering (Yang et al., 2019; Collazos-Huertas et al., 2020; Cury et al., 2020). In this paper, to overcome the signal distortion effect in PLA removal of EEG signals, we propose a novel approach based on the combination of SR and spectrum correction (SC). SC methods can identify the harmonic parameters based on the information in the frequency domain. It will be shown in this paper that for a sinusoidal component, the SC can be regarded as a numerical algorithm to realize SR *via* a non-iterative way. In ratio-based spectrum correction (RBSC) algorithms, the performance of harmonic parameter extraction is determined by signal-to-noise ratios of baseline spectral lines (BSSLs). Taking this phenomenon into account, a dual-step correcting algorithm (DSCA) is put forward. Firstly, a RBSC, using the rectangular window, is employed for preliminary identification of harmonic parameters. Secondly, two BSSLs with high SNR are calculated. Thirdly, another RBSC is employed to estimate the harmonic parameters with higher precision. On the basis of the estimated harmonic parameters, a compensation signal is reconstructed. PLA removal by subtracting the compensation signal from the original EEG signal can avoid the side effect of signal distortion. Analysis using numerical simulation and measured signal shows that the proposed method is effective, efficient, and can suppress waveform distortion.

## Materials and Methods

### Mechanism of Signal Distortion in Electroencephalogram Pre-processing

**FIGURE 1:**
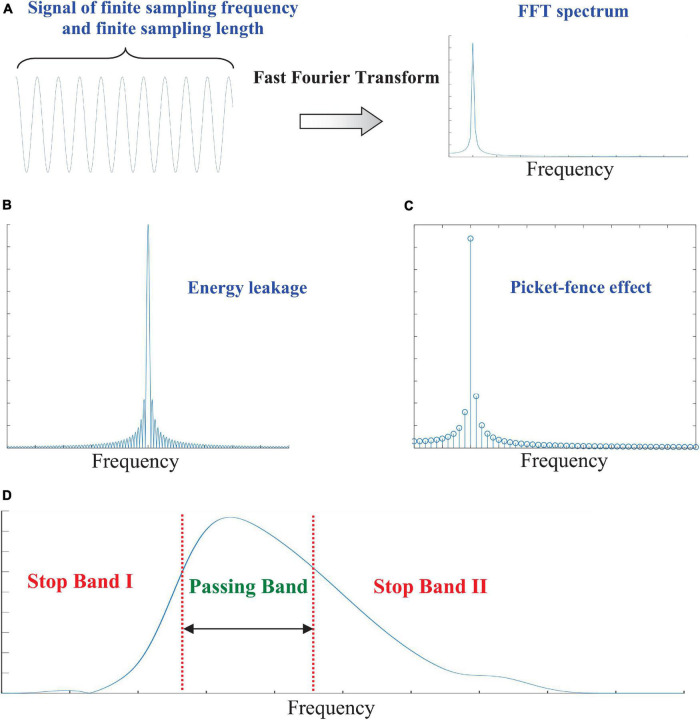
**(A)** Digitization of dynamic processes with finite sampling frequency and a finite sampling number; **(B)** FFT spectrum of a sinusoidal component; **(C)** the energy leakage problem; **(D)** the picket-fence effect (PFE) and the filtering characteristics of a digital filters from Daubechies wavelet basis (Mallat, 1989; Chen et al., 2012).


To ensure high performance of BCI, noises in EEG signals should be suppressed using pre-processing based on hardware-based or software-based solutions. However, severe signal distortions may be produced by such pre-processing. The theory of Fourier transform can be used to explain the reason of this side effect. An EEG signal*x*(*t*), corrupted by the PLA, can be expressed as*x*(*t*) = *eeg*(*t*) + *pla*(*t*), where *eeg*(*t*) and *pla*(*t*) stands for the salient EEG features and PLA, respectively. In most cases, the PLA component can be modeled as a sinusoidal component, expressed as*pla*(*t*) = *A*_*pla*_ ⋅ *cos*(2π*f*_*pla*_*t* + φ_*pla*_). These harmonic parameters (*A*_*pla*_,*f*_*pla*_,φ_*pla*_) are crucial to determine the waveform of PLA. In actual analysis of a dynamic process, the digitization of a physical quantity requires the signal to have a finite sampling frequency (*f*_*s*_) and a finite sampling number (*N*) (Figure 1A). That is to say, after digitization, *x*(*t*) should be expressed as *x*[*n*] = *x*((*n* − 1) ⋅ Δ*t*), where *n* = 0, 1, …, *N* − 1 and the spatial interval is denoted as Δ*t* = 1/*f*_*s*_. The FFT converts an input signal from the time domain into a counterpart in the frequency domain. In numerical implementations of FFT, a cluster of sinusoidal waves are used to decompose the input signals.

(1) X[ℓ]=FFT{x[n]},

where *X*[ℓ] (ℓ = 0, 1 …, *N* − 1) represents the complex-valued coefficient at the frequency of (ℓ − 1) ⋅ *f*_*s*_/*N*. The FFT spectrum is also a sampling of actual Fourier spectrum of *x*(*t*). If the frequency *f*_*pla*_ does not belong to the set {(ℓ − 1) ⋅ *f*_*s*_/*N*| ℓ = 0, 1 …, *N* − 1}, the problem of energy leakage and PFE occur (Chen et al., 2019). In such circumstance, the FFT spectrum of {*pla*[*n*]} is dense in frequency domain (Figure 1B). The energy of the noisy component {*pla*[*n*]} leaks across the entire frequency domain. A simple harmonic wave in time domain becomes a broad band signal in the frequency domain. Therefore, the harmonic parameters cannot be estimated using any single spectral line in the FFT spectrum (Figure 1C).

Wavelet transform and EMD are important developments of the classical FFT. However, they are essentially digital filters with specific passing band in the frequency domain (Figure 1D). By using them, only a part of the PLA spectrum can be suppressed. The residual contents after digital filtering still leads to side effect of signal distortion. This phenomenon is similar to Gibbs phenomenon in Fourier analysis. Therefore, it can also be summarized as a pseudo-Gibbs phenomenon (PGF) in multi-scale analysis.

### Fundamentals of Sparse Representation

Sparse representation has become a hot research topic in the field of signal processing during the past two decades. SR aims at expressing an input signal *x* as a linear combination of atoms from an over-complete dictionary Φ.


(2) $$x=\Phi c=\left[\begin{array}{cccc} \varphi_{1} & \varphi_{2} & \cdots & \varphi_{M} \end{array}\right]\left(\begin{array}{c} c_{1}\\ c_{2}\\ \vdots \\ c_{M} \end{array}\right),$$

where φ_*i*_ ∈ *Φ* (*i* = 1, 2, …, *M*) and *M* is the number of atoms in Φ. For EEG signal analysis, the variables *x* and φ_*i*_ are supposed to be column vectors of dimension *K*. The dimension of Φ in matrix form is *K* × *M*. Different from a basis, the condition *M* × *K* should be satisfied for an SR dictionary Φ. To satisfy the demand of sparsity, most coefficients in the set {*c*_*i*_} are approximate or equal to zero.

To obtain a sparse solution $\hat{c}$, we need to solve the following optimization problem.


(3) $$\hat{c}=\mathrm{argmin}∥c{∥}_{0},\;s.t.\;x=\Phi c$$

where the L0-norm ||*c*||_0_ calculates the number of non-zero terms in $\hat{c}$. However, this problem has proven to be NP-hard. To deal with this problem, the L1-norm ${||c||}_{1}=\sum_{i=1}^{K}\left|c_{i}\right|$ can be introduced to derive a feasible solution. Therefore, the problem in Eq. (3) is formulated as below.


(4) $$\hat{c}=\mathrm{argmin}∥c{∥}_{1},\;s.t.\;x=\Phi c$$

In practical applications, owing to the existence of measurement noises, the constraint condition *x* = Φ*c* is usually too strict that may results in no solution. A relaxed L2-norm constraint, allowing a maximum error of ε, is more convenient.


(5) $$\hat{c}=\mathrm{argmin}∥c{∥}_{1},\;s.t.\;∥x-\Phi c{∥}_{2}\leq \text{ε}$$

To solve the above optimization problem, iterative algorithms, such as matching pursuit and basis pursuit, can be utilized. From the above argument, the solution of $\hat{c}$ depends on the choosing of a proper dictionary Φ. Therefore, a critical problem of SR in engineering applications lies in the selection a proper over-complete dictionary.

### Spectrum Correction for Sinusoidal Component

Fast Fourier transform is extensively applied to investigate the component of *x*(*t*) = *A*_*c*_ ⋅ *cos*(2π*f*_*c*_*t* + φ_*c*_), which appears as a simple harmonic wave in a dynamic process. According to the fundamentals of digital signal processing, if the condition *f*_*s*_/*f*_*c*_ ∈ *N*^+^ is satisfied, the information of the PLA noise can be uniquely revealed by one spectral line, whose frequency is exactly*f*_*c*_, in the FFT spectrum. However, this condition is difficult to meet in most cases because the value of *f*_*c*_ can be a complicated decimal. In such circumstances, SCs, based on the information in FFT spectra, should be adopted. In this section, we take the rectangular window function as an example to illustrate the SC algorithm.

In FFT analysis, the spectral resolution is defined as Δ*f* = 1/(*N* ⋅ Δ*t*). Spectral lines are uniformly spaced by one single spectral resolution in the frequency axis. According to the algorithm of RBSC, a pair of spectral lines whose frequencies differ by one spectral resolution are utilized to estimate the harmonic parameters. For ease of argument, we name the two spectral lines as BSSLs. The frequencies of BSSL are denoted by *f*_*l*_ and *f*_*r*_, and they are belonging to the spectral grid set {(ℓ − 1) ⋅ *f*_*s*_/*N*| ℓ = 0, 1 …, *N* − 1}. To estimate harmonic parameters of a sinusoidal component at the frequency of *f*_*c*_, the following relationship is satisfied.


(6) $$f_{c}-0.5\leq f_{l}<f_{c}<f_{r}\leq f_{c}+0.5$$

**FIGURE 2:**
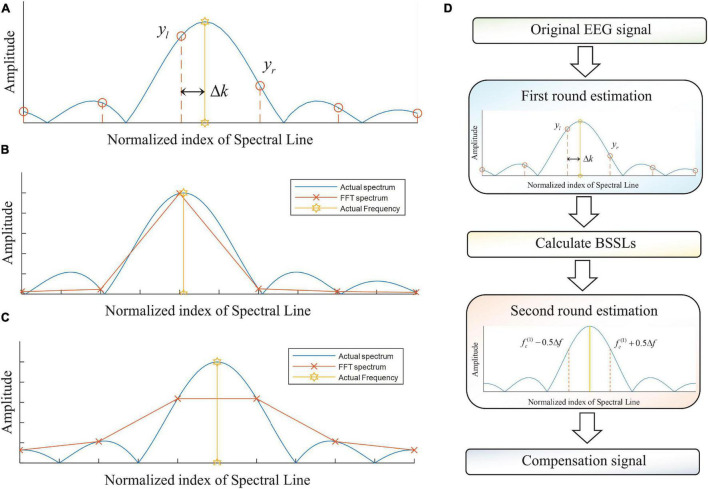
**(A)** Mathematical model for the RBSC; **(B)** RBSC for small normalized frequency error; **(C)** RBS for normalized frequency error Δ*k* = 1/2; and **(D)** the flowchart of the DBSC.


The concept of normalized index of spectral line is used to indicate the frequency of a spectral line in FFT spectrum. Let *Y*_*k*_ be the complex number associated with the spectral line whose frequency is (*k* − 1) ⋅ Δ*f*, *y*_*k*_ and *arg*(*Y*_*k*_) can be used to represent amplitude and argument of the *Y*_*k*_. Figure 2A shows the amplitudes (*y*_*l*_ and *y*_*r*_) of BSSLs in RBSC. The normalized frequency shift is calculated by Δ*k* = (*f*_*c*_ − *f*_*l*_)/Δ*f*. In RBSC, the ratio between *y*_*l*_ and *y*_*r*_ can be used to calculate Δ*k* conveniently, which is implemented based on the information fusion of BSSLs. An indicator of characteristic ratio for the SC can be defined as *R* = *y*__*l*_/*y*_*r*_. Using the characteristic ratio, the normalized error Δ*k* can be calculated as below.

(7) $$\Delta k=-\frac{1}{1+R}=-\frac{y_{k}}{y_{k}+y_{k+1}}$$

Then the estimated harmonic parameters (${\tilde{A}}_{c}$, ${\tilde{f}}_{c}$, and ${\tilde{\varphi }}_{c}$) of the investigated sinusoidal component can be derived as below.


(8) $$\left\{ \begin{array}{c} {\tilde{A}}_{c}=\frac{\pi \cdot \Delta k\cdot y_{k}}{\sin\left(\pi \cdot \Delta k\right)}\\ {\tilde{f}}_{c}=\left(k+\Delta k\right)\cdot \Delta f\\ {\tilde{\varphi }}_{c}=\arg\left(Y_{k}\right)-\pi \cdot \Delta k \end{array}\right.$$

The sinusoidal component is supposed to be produced using the estimated harmonic information. In summary, the RBSC produces a harmonic atom that is very similar to the original PLA. This harmonic atom can be treated as a special dictionary with *M* = 1<< *K*. This is the relationship between SC and SR for sinusoidal component analysis.

### The Proposed Method

The RBSC is an effective method to estimate harmonic parameters of a sinusoidal component. It utilizes information of BSSLs in FFT spectrum to estimate the harmonic parameters. In actual dynamic measurements, the information of BSSLs is also affected by noises. Therefore, high SNR are important to ensure estimations of high precision. As the value of Δ*k* approaches zero, the SNR of *y*_*r*_ reduces significantly if measurement noise exists (Figure 2B). As a result, significant estimation errors can be produced for the harmonic parameters. As a compromise, we expect Δ*k* to be approximately 1/2 such that both of *y*_*l*_ and *y*_*r*_ are of high amplitudes to resist the measurement noises (Figure 2C). To address this problem, a DSCA is proposed. The key idea of DSCA is the numerical implementation of spectral correction using BSSLs of high amplitudes. The flowchart of the DBSC is shown in Figure 2D.

Step 1. Apply FFT for the input signal *x*(*t*) = *A*_*c*_ ⋅ *cos*(2π*f*_*c*_*t* + ϕ_*c*_).

(9) $$x\left(t\right)\overset{FFT}{⟶}X\left(k\right)$$

Step 2. First round estimation of harmonic parameter in *x*(*t*) using RBSC.

(10) $$X\left(k\right)\overset{RBSC}{⟶}A_{c}^{\left(1\right)},f_{c}^{\left(1\right)},\varphi_{c}^{\left(1\right)}$$

Step 3. Calculate BSSLs at frequencies of $f_{c}^{\left(1\right)}-0.5\cdot \Delta f$ and $f_{c}^{\left(1\right)}+0.5\cdot \Delta f$ based on $f_{c}^{\left(1\right)}$ and the FFT spectrum.

(11) $$\left\{ \begin{array}{c} f_{c}^{\left(1\right)}\\ X\left(k\right) \end{array}\right.\overset{}{⟶}\left\{ \begin{array}{c} f_{c}^{\left(1\right)}-0.5\cdot \Delta f\\ f_{c}^{\left(1\right)}+0.5\cdot \Delta f \end{array}\right.$$

Step 4. Second round estimation of the harmonic parameters (${\tilde{A}}_{c},{\tilde{f}}_{c},{\tilde{\varphi }}_{c}$) using RBSC.

(12) $$\left\{ \begin{array}{c} f_{c}^{\left(1\right)}-0.5\cdot \Delta f\\ f_{c}^{\left(1\right)}+0.5\cdot \Delta f \end{array}\right.\overset{RBSC}{⟶}A_{c}^{\left(2\right)},f_{c}^{\left(2\right)},\varphi_{c}^{\left(2\right)}$$

Step 5. The harmonic parameters are corrected as below.

(13) $$\left\{ \begin{array}{c} {\tilde{A}}_{c}=A_{c}^{\left(1\right)}\\ {\tilde{f}}_{c}=f_{c}^{\left(2\right)}\\ {\tilde{\phi }}_{c}=\varphi_{c}^{\left(2\right)} \end{array}\right.$$

Step 6. A compensation signal can be produced using the corrected harmonic information.

(14) $$\tilde{x}\left(t\right)={\tilde{A}}_{c}^{\left(1\right)}\cdot \cos\left(2\pi {\tilde{f}}_{c}^{\left(2\right)}t+{\tilde{\varphi }}_{c}^{\left(2\right)}\right)$$

According to the above algorithm, no iteration is employed. The original sinusoidal component is retrieved with errors $∥x\left(t\right)-\tilde{x}\left(t\right)∥>0$. Only one atom, learned by the spectral corrections, is used to reconstruct *x*(*t*). This can be seen as a special form of SR for sinusoidal component.

## Results

### Numerical Simulations

**FIGURE 3:**
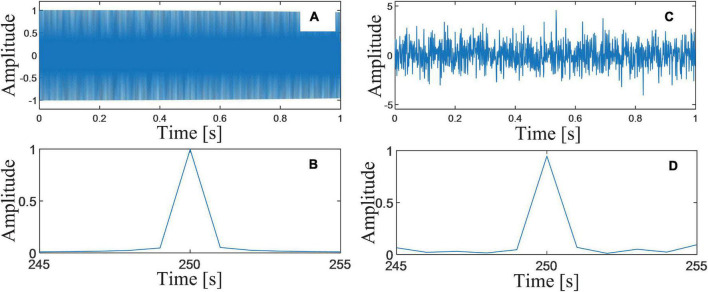
**(A)** Waveform of the simulated PLA; **(B)** FFT spectrum of the simulated PLA; **(C)** the simulated PLA with noises (SNR = 0dB); and **(D)** FFT spectrum of the simulated PLA with noises.

To verify the enhancement of the proposed DSCA, numerical simulations are employed in this sub-section. A signal, consisting of PLA (*pla*(*t*), Figure 3A) and noises (*n*(*t*)), is simulated. Without loss of generality, the harmonic parameters are set as *A*_*pla*_ = 1, *f*_*pla*_ = 250.05*Hz*, and φ_*pla*_ = 0. A white Gaussian noise *n*(*t*) is added to the simulated signal, and the SNR is set as 0dB (Figure 3C). Both of the sampling frequency and the sampling number are set as 1,000. The spectral resolution of the signal in spectral domain is calculated as Δ*f* = 1 (Figures 3B,D). In FFT spectrum of *pla*(*t*), the frequencies of BSSLs are 250 and 251 Hz. Their amplitudes are *y*_*l*_ = 0.996 and *y*_*r*_ = 0.052. The normalized frequency shift is Δ*k* = 0.05, which is very closed to zero. The harmonic parameters are corrected as *A*_*pla*_ = 1, *f*_*pla*_ = 250.05*Hz*, and ϕ_*pla*_ = 0. The estimation accuracy is perfect when there is no noise. While in the spectrum of *s*(*t*), *y*_*l*_ = 0.947 and *y*_*r*_ = 0.068. The harmonic parameters are corrected as *A*_*pla*_ = 0.9539, *f*_*pla*_ = 250.067*Hz*, and ϕ_*pla*_ = −0.010. It can be seen significant estimation errors occur due to noises.

For the proposed DSCA, the first-round estimation of the frequency is $f_{c}^{\left(1\right)}=250.067$. The frequencies of BSSLs are *f*_*l*_ = 249.567 and *f*_*r*_ = 255.067. The harmonic parameters are corrected as *A*_*pla*_ = 1.073, *f*_*pla*_ = 250.040*Hz*, and φ_*pla*_ = −0.006. It can be seen that the estimation errors of the frequency and the phase are reduced significantly, while estimation error of the phase increases. The same rule of error has also been verified in other independent repeated experiments. That is the reason why we use the estimated harmonic parameters in different rounds of estimations.


(15) s(t)=pla(t)+n(t)

### Case Study of Noisy Electroencephalogram Signals

**FIGURE 4:**
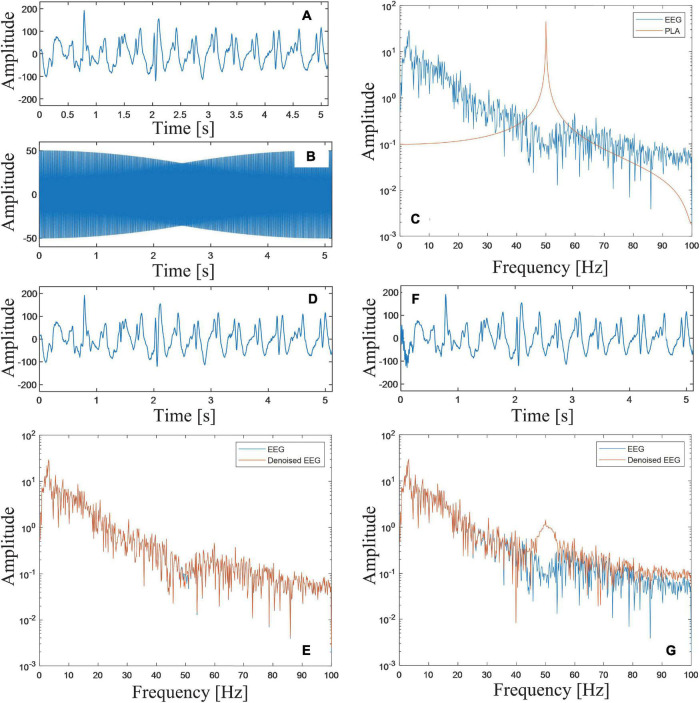
**(A)** Time domain waveform of the EEG signal; **(B)** the simulated PLA noise; **(C)** FFT spectra of the EEG signal and the PLA noise; **(D)** the denoised signal based on the proposed DSCA; **(E)** FFT spectra of the EEG signal and the filtered signal using DSCA; **(F)** the denoised signal based on DNF method; and **(G)** FFT spectra of the EEG signal and the filtered signal using DNF method.

In this sub-section, actual measurements of EEG signals are employed to further verify the performance of the proposed DSCA. The EEG datasets, we used in this paper, were made available to the public by Neurology & Sleep Centre, Hauz Khas, New Delhi. The employed datasets contain EEG records from clinical studies concerning epilepsy. A record of EEG time series is selected from the datasets (Figure 4A). The sampling frequency is 200 Hz, and the sampling number is 1,000. In acquisition of the EEG time series, a band-pass digital filter with the passing band of [0.5, 70]*Hz* was applied. To simulate the PLA, an additive noisy component is synthesized with the parameters *A*_*pla*_ = 50, *f*_*pla*_ = 250.05*Hz*, and φ_*pla*_ = 0(Figure 4B). The FFT spectra of the two components are shown in Figure 4C. A noisy EEG signal is formed by directly superimposing the two components. The SNR of the investigated signal is 3.430 dB.

The proposed DSCA is applied to remove the PLA. The harmonic parameters are corrected as *A*_*pla*_ = 50.006, *f*_*pla*_ = 250.050*Hz*, and φ_*pla*_ = −0.023. Using these estimated parameters, the compensation signal can be reconstructed and subtracted from the synthesized signal. The denoised signal is plotted in Figure 4D. From the appearances of the two waveforms, they are very similar. The correlation coefficient of the two signals are computed as 1.00, indicating that the filtering result is satisfactory. The FFT spectra of the denoised signal and the original noisy EEG signal are shown in Figure 4E. The difference only exists in a very narrow frequency band around 50 Hz.

As comparison, a DNF based method is also used to process the noisy signal (Chen et al., 2012). The denoised signal is shown in Figure 4F. In the time domain waveform, there is a severe distortion on the left side of the filtered signal. The correlation coefficient of the two signals are computed as 0.997. While, from the frequency domain (Figure 4G), differences between the two signals increase significantly near 50 Hz. The above comparisons show that the proposed DSCA algorithm in this paper outperforms DNF in PLA removal.

## Discussion

The problems of energy leakage and PFE are inevitable for harmonic components whose digital samplings do not satisfy the full period sampling condition (Mewett et al., 2001). There is a view that these two side effects can be prevented by actively adjusting the sampling parameters. Because a variation of ± 2 Hz is likely to occur in actual power systems (Singhal et al., 2020), the parameter *f*_*pla*_ is an unknown variable. It is not practical to design DNFs perfectly suitable for all possible PLA noises. On the other hand, increasing the sampling length of EEG signal can improve the accuracy of harmonic parameter estimation. However, the process of brain electrical activity corresponding to the collected signals cannot be repeated, so we can only analyze and process the signals of limited length.

Wavelet transform and EMD outperform FFT by providing time-frequency representation of EEG signal. However, in either case, the above side effect is still unavoidable. Because no matter how they decompose the signal, they can only suppress part of the PLA, and the rest will still cause signal distortion. In principle, RBSC uses local spectral information to estimate the overall harmonic composition, so it can avoid the side effect.

The method proposed in this paper is an improvement of the classical RBSC. It not only improves the robustness of the algorithm in parameter correction, but also considers the efficiency of the algorithm. The complexity of the algorithm has only a small increase compared with the classical method. The method proposed in this paper is especially suitable for the case that the value of Δ*k* is very small. When the value of this parameter is close to half the frequency resolution, the performance of the two methods is close. In general, the method proposed in this paper can be better applied to the complex measurement environment. However, in any case, the method in this paper has no less accuracy than the original RBSC in statistics. So we recommend that DSCA can completely replace the original RBSC.

Ratio-based spectrum correction only utilize two BSSLs to estimate harmonic parameters, and it can balance the efficiency and accuracy of the algorithm. In the literature, there have been some methods for harmonic parameter estimation using multiple BSSLs. These studies also show that their harmonic parameter estimation accuracy is indeed better than that of RBSC in the absence of noise. But this is not achievable in clinical applications. Therefore, the DSCA is proposed based on the RBSC. The calculation time of RBSC mainly includes the FFT transform of the signal and several complex arithmetic operations. The DSCA proposed in this paper mainly includes two RBSCs, so the efficiency is also very high.

For analysis of a simple harmonic component, the spectral correction is very similar with the spare representation theory in mathematical principle. A remarkable advantage is that it does not require a predetermined dictionary. The algorithm can generate a skinny dictionary containing only one atom to represent the actual sinusoidal component. Because the dictionary is not redundant, no iterative algorithms is needed. We can call the spectral correction as implicit sparse representation (ISR) for simple harmonic component analysis. The current research shows that the results obtained by using SR based on iterative numerical algorithm are very similar to those based on spectral correction method. Therefore, in the case of harmonic analysis, a method based on SC may be preferentially employed.

## Data Availability Statement

The data presented in the study are deposited in the ictal repository, accession number ictal1 (https://www.researchgate.net/publication/308719109_EEG_Epilepsy_Datasets).

## Author Contributions

MZ conceived and designed the classification method. Z-FL, RZ, and NX performed the experiment. Z-FL, RZ, Y-QD, and G-HL analyzed the data and wrote the manuscript. MZ and NX reviewed and edited the manuscript. All authors read and approved the manuscript.

## Conflict of Interest

J-LT, Z-FL, and RZ were employed by the company Shaanxi Aerospace Technology Application Research Institute Co., Ltd. The remaining authors declare that the research was conducted in the absence of any commercial or financial relationships that could be construed as a potential conflict of interest.

## Publisher’s Note

All claims expressed in this article are solely those of the authors and do not necessarily represent those of their affiliated organizations, or those of the publisher, the editors and the reviewers. Any product that may be evaluated in this article, or claim that may be made by its manufacturer, is not guaranteed or endorsed by the publisher.
